# Production of infectious HCV genotype 1b virus in cell culture using a novel Set of adaptive mutations

**DOI:** 10.1186/s12866-016-0846-9

**Published:** 2016-09-27

**Authors:** Ken-ichi Mori, Akihiro Matsumoto, Noboru Maki, Yuki Ichikawa, Eiji Tanaka, Shintaro Yagi

**Affiliations:** 1 R&D Department, Advanced Life Science Institute, Inc., 2-10-23 Maruyamadai, Wako, Saitama 351-0112 Japan; 2 Department of Medicine, Division of Hepatology and Gastroenterology, Shinshu University School of Medicine, 3-1-1 Asahi, Matsumoto, Nagano 390-8621 Japan

**Keywords:** HCV, HCVcc, Genotype 1b, NS2, NS4B, Adapted mutation

## Abstract

**Background:**

Despite the high prevalence of genotype 1b hepatitis C virus (HCV) among patients, a cell culture system that permits entire viral life cycle of genotype 1b isolates is limited. To develop a cell-cultured hepatitis C virus (HCVcc) of genotype 1b, the proper combination of HCV genomic variants and host cells is essential. HCV genomes isolated from patients with distinctive symptoms may provide the variants required to establish an HCVcc of genotype 1b.

**Results:**

We first established subgenomic replicons in Huh7 cells using HCV cDNAs isolated from two patients: one with fulminant hepatitis after liver transplantation (TPF1) and another with acute hepatitis and moderate symptoms (sAH). Replicons established from TPF1 and sAH showed mutations in NS4B and in NS3 and NS5A, respectively. Using these replication machineries, we constructed HCV genomic RNAs for each isolate. Virus infectivity was evaluated by a focus-forming assay, which is dependent on the intracellular expression of core antigen, and production of virus particles was assessed by density-gradient centrifugation. Infectious virus was only observed in the culture medium of cells transfected with TFP1 HCV RNA. A chimeric genome with the structural segment (5′-untranslated region [UTR] through NS2) from sAH and the replication machinery (NS3 through 3′-UTR) from TPF1 exhibited greater infectivity than did TFP1, despite formation of deficient virus particles in sAH, suggesting that this genomic segment potentiates virus particle formation. To identify the responsible variants, infectious virus formation was assessed in a chimeric genome carrying parts of the sAH structural segment of the TPF1 genome. A variant in NS2 (M170T) was identified that enhanced infectious virus formation. HCVcc carrying an NS2 gene encoding the M170T substitution and adaptive mutations in NS4B (referred to as TPF1-M170T) infected naïve cured Huh7 cells in a CD81-dependent manner.

**Conclusions:**

We established a novel HCVcc of genotype 1b in Huh7 cells by introducing an amino acid variant in NS2 and adaptive mutations in NS4B from HCV genomic RNA isolated from a patient with fulminant HCV after liver transplantation.

**Electronic supplementary material:**

The online version of this article (doi:10.1186/s12866-016-0846-9) contains supplementary material, which is available to authorized users.

## Background

The hepatitis C virus (HCV) chronically infects approximately 130–150 million people annually worldwide, and 350,000–500,000 deaths every year are attributed to HCV–related liver diseases (World Health Organization web site, 2015: http://www.who.int/mediacentre/factsheets/fs164/en/). The genome of HCV, which belongs to the Flaviviridae family, comprises single-stranded RNA of about 9.6 kb consisting of untranslated regions (UTRs) at each end and a long open reading frame (ORF). The ORF is translated from an internal ribosome entry site (IRES) to generate structural (core, E1 and E2) and non-structural (p7, NS2, NS3, NS4A, NS4B, NS5A, and NS5B) proteins [1]. HCV has been classified into seven major genotypes and several subtypes. In particular, genotype 1 (subtypes 1a and 1b) is responsible for the majority of known HCV infections and is resistant to pegylated interferon (PEG-IFN) and ribavirin (RBV) therapy [2, 3]. In Japan, triple-combination therapy for chronic HCV involving protease inhibitors and PEG-IFN/RBV has been approved for treatment of infections with the major HCV subtype (1b) since 2011.

The establishment of HCV subgenomic replicons was an important advancement for virological HCV research, leading to the development of direct-acting antiviral drugs [4]. This in turn resulted in a second major breakthrough with the establishment of cell-cultured HCV (HCVcc) from an HCV clone (JFH-1) isolated from a patient with fulminant hepatitis C [5]. The JFH-1 HCVcc was shown to infect Huh7 cells in a CD81-dependent manner, and could self-replicate using its own NS5B RNA-dependent RNA polymerase (RdRp). HCVcc completes its entire life cycle in vitro, and chimeric HCVcc strains harboring structural segments (the core through NS2) from the HCV genomes of all seven genotypes and the JFH-1 replicon have been developed [5–10].

Adaptive mutations that improve the efficiency of viral replication have been identified in replication systems using these subgenomic replicons and HCVcc strains [11–17]. Mutations located between the NS3 and NS5A proteins mostly enhance the replication of genomic RNA. In addition to these mutations in the viral genome, mutations in host cells, such as those found in Huh7.5 cells, which are Huh7 cells that have had the subgenomic replicons removed by IFN treatment, show great impacts on not only genomic replication but also infectious virus formation [7, 15, 18].

The use of HCVcc has enabled the identification of mutations in the p7 and NS2 proteins, which affect the assembly of infectious virus [14, 19]. The effects of these mutations were also shown to be enhanced by other mutations in NS3 and NS5A, suggesting cross-talk between these HCV proteins [13, 14, 16, 17]. The p7 and NS2 proteins are indispensable for infectious virus formation in trans-packaging systems as well as HCVcc [20]. The direct or indirect interaction of these proteins with structural proteins such as the capsid protein, core, and membrane proteins E1/E2 was suggested by their subcellular localization [21–23]. In addition, compensatory mutations for virus assembly defects caused by mutations in the core protein were identified in p7 and NS2 [24].

Combinations and applications of these variants has improved the development of HCVcc using JFH-1 and Huh7.5 cells, and has helped to establish HCVcc genotypes 1a, 2a, 2b, and 3a [25–30]. Although subgenomic replicons of genotype 1b were established at an early stage in HCV research, no robust HCVcc genotype 1b has been reported to date because of interference with virus particle formation by the adapted mutations in the replicons [31, 32]. As described above, the genomic context, i.e., the combination of variants, including adaptive mutations in the genome, is an important consideration for virus replication. Therefore, we hypothesized that the proper combination of variants in genotype 1b would enable successful establishment of an HCVcc of genotype 1b. In this study, we isolated HCV cDNA from two patients infected with genotype 1b HCV and identified the variants that would best allow for replication of genotype 1b HCVcc in Huh7 cells.

## Methods

### Ethics Statement

This study was approved by the ethical committee of Shinshu University School of Medicine, Matsumoto, Japan, and written informed consent was obtained from all patients. The study was conducted in accordance with the principles of the Declaration of Helsinki.

### Cell culture

The human hepatocarcinoma cell line Huh7 (JCRB0403) and Huh7-derived cured cells were cultured in 5 % CO_2_ at 37 °C in Dulbecco’s modified Eagle medium (DMEM) supplemented with 10 % fetal bovine serum, 50 U/mL penicillin, and 50 μg/mL streptomycin.

### Cloning of HCV cDNA

Serum specimens were obtained from patients at Shinshu University Hospital. Total RNA was recovered from the samples using the High Pure Viral RNA kit (Roche) according to the manufacturer’s instructions. HCV cDNA was amplified by long-distance RT-PCR as described previously [33–35]. HCV cDNA was synthesized using Superscript II reverse transcriptase (Invitrogen) with the XR58R primer. After RNase H (Takara) treatment at 37 °C, the cDNA mixture was amplified by PCR with LA Taq DNA polymerase (Takara) and the HC-Long A1 and 1b5290AS primers, or the HC4498S and 1b9405R primers for 30 cycles of denaturation at 94 °C for 20 s and extension at 68 °C for 5 min. A second-round PCR was performed with the HC85F and 1b5290AS primers or the HC4888F and HC9302R primers for 20 cycles under the same conditions as the first-round PCR. The PCR products were purified from the gel using the QIA-quick gel extraction kit (QIAGEN), and were then cloned into the pGEM-T Easy vector (Promega). The cDNA clones TPF1-0153 and TPF1-4893 were amplified from serum samples from patient TPF1, and sAH-0153 and sAH-4893 were obtained from serum samples from patient sAH.

Four clones of each fragment were sequenced with a CEQ-2000XL DNA analysis system (Beckman Coulter) with a DTCS quick start kit and HCV-specific primers according to the manufacturer’s instructions. Sequence data were analyzed with Sequencher (Gene Codes Corporation) and MacVector (MacVector, Inc.) software packages.

HCV cDNA of the 5′UTR was obtained by 5′ rapid amplification of cDNA ends (RACE), which was carried out using a 5′RACE System (v. 2.0; Invitrogen) according to the manufacturer’s instructions. HCV RNA was reverse-transcribed from serum samples with the chiba-AS primer and subjected to a TdT-tailing reaction. PCR was carried out with the 5′RACE Abridged Anchor primer and the KY78 primer. A second-round PCR was carried out with the Universal Amplification and KM2 primers, and cloned into the pGEM-T easy vector. HCV cDNA clones containing the nucleotides 1–709 were designated TPF1-0007 and sAH-0007.

To obtain the 3′UTR cDNA of TPF1 and sAH (TPF1-8994 and sAH-8994, respectively), cDNA was synthesized with the XR58R primer, and then amplified by PCR with the HC8939F and XR58R primers. The terminus of the 3′UTR cDNA was obtained by 3′RACE. Poly (A) tails were added to the total RNA in the serum samples from patients TPF1 and sAH using the Poly (A) Tailing Kit (Ambion) according to the manufacturer’s instructions. HCV cDNA was synthesized with the dT-Adp primer and then amplified by PCR with the 3UTR-1 F and Adp primers. A second-round PCR was carried out with primers XR58F and Adp, and then cloned (TPF1-3′UTR and sAH-3′UTR). The primers used for this study are shown in Additional file 1: Table S1.

### Plasmid construction

In order to construct full-length HCV plasmids, PCR was carried out with the primers 3′UTRcla and RP1xba, using two PCR products, TPF1-3′UTR and sAH-3′UTR, as the template, and the resulting cDNA clones were named TPF1-3′UTRxba and sAH-5′UTRxba, respectively. Based on the consensus sequence of TPF1, we assembled full-length HCV cDNAs containing fragments TPF1-0007, −0153, −4893, −8994, and −3′UTRxba, and the resulting construct was named TPF1con. Another full-length HCV cDNA, sAHcon, was also constructed using fragments sAH-0007, −0153, −4893, −8994, and −3′UTRxba. The cDNA encoding TPF1con and sAHcon was inserted downstream of the T7 RNA promoter in pBluescript SKII(+) (Agilent Technologies) to construct TPF1 (DDBJ/EMBL/GenBank accession number LC011927) and sAH (accession number LC011930), respectively. The TPF1 subgenomic replicon (rep_TPF1) and the sAH subgenomic replicon (rep_sAH) constructs were obtained by replacing the regions encoding the structural proteins and the NS2 protein with the sequences encoding neomycin phosphotransferase (Neo) from a UAS-probe plasmid [36] and the encephalomyocarditis virus (EMCV) IRES as described previously [11].

### Subgenomic replicon assay

*Xba*I-linearized plasmids encoding the TPF1 and sAH replicons were in vitro-transcribed by T7 RNA polymerase with the MEGAscript T7 kit (Ambion). The synthesized replicon RNAs were purified according to the manufacturer’s instructions and then electroporated into Huh7 cells. In brief, 10 μg of replicon RNA was mixed with 4 × 10^6^ Huh7 cells in a 4-mm cuvette and then pulsed at 250 V and 950 μF using Gene Pulser (Bio-Rad). Replicon RNA-transfected cells were suspended in 8 mL of complete DMEM culture medium and transferred into a 100-mm culture dish (Iwaki). After 24 h of incubation, G418 (Neomycin) was added to the culture medium (at 0.25–1 mg/mL). The medium was changed every 4 days, and 20 days after electroporation, the surviving cell clones were stained with PhastGel blue R (Sigma-Aldrich) or cloned and expanded by growth in individual plates.

### Sequence analysis of G418-resistant cells

Total RNA was extracted from the cloned G418-resistant cells using the High Pure RNA Isolation kit (Roche) according to the manufacturer’s instructions, and the cDNAs of the replicon RNA were synthesized with the XR58R primer. PCR amplification was performed with the EMCV-S1 and 1b9405R primers. The sequence of each recovered replicon, rep_TPF1 and rep_sAH, was determined. Plasmids for replicons rep_TPF1-4B and rep_sAH-N3.5 containing the adaptive mutations were generated by site-directed mutagenesis of the original constructs, which were identified in the recovered replicons, using the Quick Mutagenesis kit (Stratagene) according to the manufacturer’s instructions.

### Construction of full-length HCV RNA

The full-length HCV construct TPF1-4B (accession number LC011928) was developed by replacing the *Sfi*I fragment with the corresponding fragment from rep_TPF1-4B, containing the cell culture adaptive mutations in NS4B (Q93L and E255K). Similarly, sAH-N3.5 (accession number LC011931) was constructed by replacing the *BsrG*I–*Sfi*I fragment of rep_sAH-4B, which contains two cell culture adaptive mutations in NS3 (T261K) and NS5A (S232R). As a control, a replication-incompetent, full-length HCV construct (TPF1-ΔGDD) was created by an in-frame deletion of the polymerase active-site motif GDD in the NS5B polymerase in TPF1-4B. Each mutation and deletion was confirmed by DNA sequencing.

### Quantification of the HCV core antigen

Core antigen levels were measured by sandwich enzyme immunoassay (EIA) as described previously [33, 37]. To quantify the core antigens in the culture medium and cell lysate, these samples were diluted to the negative control serum before pretreatment.

### Quantification of HCV RNA by RT-PCR

HCV RNA was recovered from the culture media and cell lysates using the High Pure Viral RNA kit (Roche) and the High Pure RNA isolation kit (Roche), respectively. For quantitative (q)RT-PCR analysis to detect the 5′UTR, HCV RNA was reverse-transcribed and amplified using the QuantiTect One-Step RT-PCR kit (QIAGEN) with primers chiba-S and chiba-AS, as described previously [33].

### Establishment of cured Huh7 cells by IFN treatment

Huh7 cells containing replicon RNA (rep_TPF1-4B) carrying adaptive mutations were cultured with human recombinant IFN-αA/D (1000 IU/mL; R&D Systems) in the absence of G418. After 3–4 days, 60 %-confluent monolayers were trypsinized, plated, and cultured for 24 h before the addition of IFN-αA/D. After 4 weeks of IFN treatment, the absence of HCV RNA was confirmed from the results of a qRT-PCR assay and sensitivity to G418. The cured cells were named ALS32.50 cells.

### Full-length HCV RNA transfection

Full-length HCV RNAs were transcribed in vitro and electroporated into cells as described above. In brief, 10 μg of in vitro-synthesized HCV RNA was mixed with 4 × 10^6^ cells and transfected into Huh7 or ALS32.50 cells. Cells were seeded into 6-well plates for HCV RNA and core Ag analyses. Virus harvests were clarified by low-speed centrifugation and passed through a 0.45-μm filter, and frozen at −80 °C.

### Sucrose density gradient analysis of virus particles

The culture medium was harvested for sucrose density gradient analysis 4 days after transfection of the full-length HCV RNA. Collected culture media were cleared by low-speed centrifugation, and passed through a 0.45-μm filter. Filtered culture media were concentrated 30-fold using an Amicon Ultra-15 device (Molecular cut-off: 1 × 10^5^ Da; Millipore). Concentrated culture media with or without NP-40 pretreatment were layered onto a stepwise sucrose gradient (10–60 %, wt/vol) and centrifuged for 16 h in a SW50.1 rotor (Beckman) at 40,000 rpm with RNase A at 4 °C. After centrifugation, about 20 fractions were collected. The HCV RNA and core antigen levels in each fraction were determined by sandwich EIA and qRT-PCR, as described above.

### Infectivity assays

Huh7 and ALS32.50 cells were seeded at 1.6 × 10^4^ cells/well in 48-well plates (Iwaki), or at 2.0 × 10^4^ cells/well in collagen-coated 8-well chamber slides (Iwaki) for 24 h before inoculation with 100 μL of filtered culture medium. The amount of core antigen in inoculated cells was determined by sandwich EIA, and the cells were tested for the presence of intracellular core antigen by indirect immunofluorescence analysis at 96 h post-infection (p.i.), as described below. Clusters of infected cells, identified by staining for core antigen, were considered as a single infectious focus, and virus titers were calculated accordingly in terms of FFU (focus-forming unit)/mL.

### Indirect immunofluorescence

Cells were fixed with methanol (99.8 %) at −20 °C for 15 min, and then stained with an anti-core monoclonal antibody (5E3) [38] at 1 μg/mL, followed by extensive washing and staining with an anti-mouse IgG labeled with AlexaFlour594 (2 μg/mL; Molecular Probes). Nuclei were counterstained with DAPI (Molecular Probes), and the slides were examined with a Zeiss Axiovert 200 M fluorescence microscope.

### Construction of full-length chimeric genomes

To construct a full-length sAH-N3.5/AgeBsr chimeric plasmid, the 5′UTR-NS2 sequences in sAH-N3.5 were replaced with the corresponding segments in the *Age*I and *BsrG*I sites in TPF1-4B. A similar strategy was used to construct sAH-N3.5/BsrXba (NS3-3′UTR), sAH-N3.5/AgeBbv (5′UTR-E2), sAH-N3.5/BbvBsr (E2-p7), sAH-N3.5/XmaBsr (NS2), sAH-N3.5/BbvNsi (p7-NS2_aa114_), and sAH-N3.5/NsiBsr (C-terminal half of NS2). Variants containing the three different amino acids sequences (Q148R, M170T, and S189L) were identified by comparing the C-terminal halves of the NS2 sequences in TPF1-4B and sAH-N3.5. Three different amino acid sequences were inserted into the TPF1-4B genome using site-directed mutagenesis to create the TPF1-Q148R, TPF1-M170T (accession number LC011929), and TPF1-S189L constructs, respectively. Variant constructs were verified by DNA sequencing.

### Neutralization assay

ALS32.50 cells were infected with culture medium supplemented with an anti-CD81 monoclonal antibody (clone JS-81; BD Pharmingen) [5] and a control monoclonal antibody (anti-HBcrAg; clone HB61) [39], or 2′C-methyladenosine (2′CMeA; Carbosynth) at final concentrations of 10 μg/mL (anti-CD81 and anti-HBsAg) or 5 μM (2′CMeA). Six hours after inoculation, the cells were inoculated into complete DMEM culture medium. Cultures were incubated for 96 h, and then the cells were harvested and lysed for sandwich EIA and qRT-PCR.

## Results

### Establishment of genotype 1b HCV subgenomic replicons from Two patients with different symptoms

**Fig. 1:**
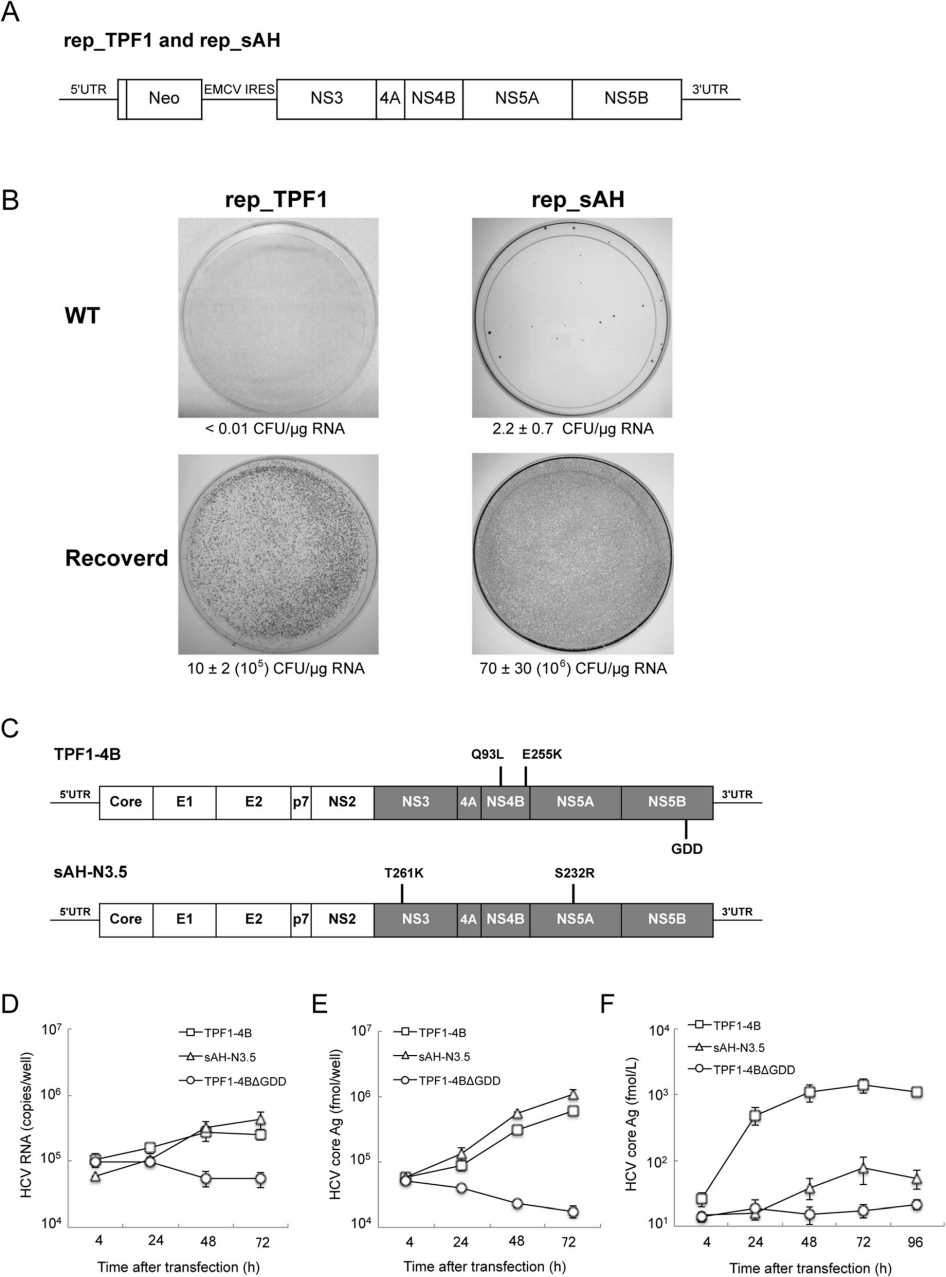
Replication of TPF1 and sAH RNAs in transfected Huh7 cells. **a** Structure of the subgenomic replicon. **b** Huh7 cells were transfected with the wild-type replicons (WT) or a replicon RNA carrying the adaptive mutations (Recovered). Numbers below the plates refer to colony-forming units (CFU) per microgram of in vitro-transcribed replicon RNA. With the rep-TPF1 of WT replicons, ten independent transfections were performed. With the rep-sAH of WT and adapted replicons, three independent transfections were performed. **c** The structures of the full-length HCV genomes TPF1-4B and sAH-N3.5 are shown. Both full-length genomes carry the heterologous sequence (*shaded area*) derived from the highly adapted subgenomic replicons, showing the location of the adaptive mutations. GDD is the active-site motif of NS5B polymerase. **d**–**f** Time course of HCV RNA replication (**d**), core Ag expression (**e**), and core Ag secretion (**f**) in transfected Huh7 cells. Huh7 cells were electroporated with TPF1-4B (□), sAH-N3.5 (△), and TPF1-ΔGDD (○) RNA transcripts. TPF1-ΔGDD contains a deletion of the polymerase active-site motif (GDD) in NS5B polymerase. The HCV RNA and core Ag levels were measured by qRT-PCR and sandwich EIA at the indicated time points, respectively. Results represent the mean of three independent experiments ± standard deviations

To identify the regions in the genotype 1b HCV genome that govern the efficiency of HCVcc replication, we isolated HCV genomes with distinctive phenotypes from two patients with different clinical symptoms, including the efficacy of IFN therapy (Table 1): patient TPF1 had recurrent fulminant hepatitis after liver transplantation, and patient sAH had acute hepatitis. The titers of the HCV specimens collected from patients TPF1 and sAH were 4 × 10^7^ copies/mL and 1.3 × 10^5^ copies/mL, respectively. Subgenomic replicons carrying the consensus sequences of TPF1 and sAH were constructed and subsequently transfected into Huh7 (JCRB0403) cells, as previously described [11]. The sAH replicon produced 2.2 ± 0.7 G418-resistant colonies per microgram of RNA, whereas the TPF1 replicon yielded a single colony when more than 100 μg of RNA was transfected (<0.01 colony per microgram of RNA) (Fig. 1a). We recovered subgenomic RNA from cells harboring the replicons and determined their nucleotide sequences. The recovered replicons, which we termed rep-sAH-N3.5 and rep-TPF1-4B, each carried two mutations: T261K (in NS3) and S232R (in NS5A) in rep-sAH-N3.5, and Q93L and E255K (both in NS4B) in rep-TPF1-4B. Both replicons exhibited significantly high colony formation efficiencies compared to that of the original construct (Fig. 1b). Therefore, these mutations were considered to be adaptive mutations that allowed for efficient replication in Huh7 cells. The positions of the mutations in rep-sAH-N3.5 were reported previously [12, 15]; however, those in rep-TPF1-4B were novel.

**Table 1:** Background information of the patients

	Patient	
	TPF1	sAH
Diagnosis	Fibrosing cholestatic hepatitis	Acute hepatitis
Genotype	1b	1b
Response of IFN treatment^a^	NR	SVR
HCV RNA in serum (copies/mL)	4.0 × 10^7^	1.3 × 10^5^
Core 70/91^b^	Mutant/Wild	Wild/Wild
ISDR (a.a.2209–2248)^c^	Wild	Wild
IRRDR (a.a.2234–2379)^d^	5	5
Subgenome^e^	Yes	No

^a^ *NR* non responder, *SVR* sustaind virologic response

^b^Pretreatment predictor of poor virologic response [51]

^c^IFN sensitivity-determining region [52]

^d^IFN/RBV resistance-determining region [53]

^e^Large in-frame deletions of E1/E2 proteins in specimens of chronically infected patients [33]

### Distinctive infectious virus-forming abilities between the Two genotype 1b HCV genomic RNAs

The full-length HCV genomic RNAs TPF1-4B and sAH-N3.5 were constructed to harbor the adaptive mutations found in the recovered subgenomic replicons (Fig. 1c). We transfected these full-length genomic RNAs along with an RNA replication-defective mutant of TPF-4B (TPF1-ΔGDD), in which the RdRp motif (Gly-Asp-Asp) in the NS5B polymerase active site was deleted, into naïve Huh7 cells, and then quantitated the intracellular HCV RNA and core antigens at several time points after transfection. The levels of RNA and core antigens in the cells transfected with TPF1-4B or sAH-N3.5 RNAs increased by 72 h post-transfection (p.t.), whereas those in cells transfected with TPF1-ΔGDD RNA decreased (Fig. 1d, e), suggesting that the increase in HCV RNA and core Ag levels occurred in an NS5B RNA polymerase-dependent manner. Although rep-sAH-N3.5 exhibited approximately 7-times higher colony-forming activity than that of rep-TPF1-4B, the HCV RNA and core antigen levels in cells transfected with TPF1-4B or sAH-N3.5 RNAs were similar.

**Fig. 2:**
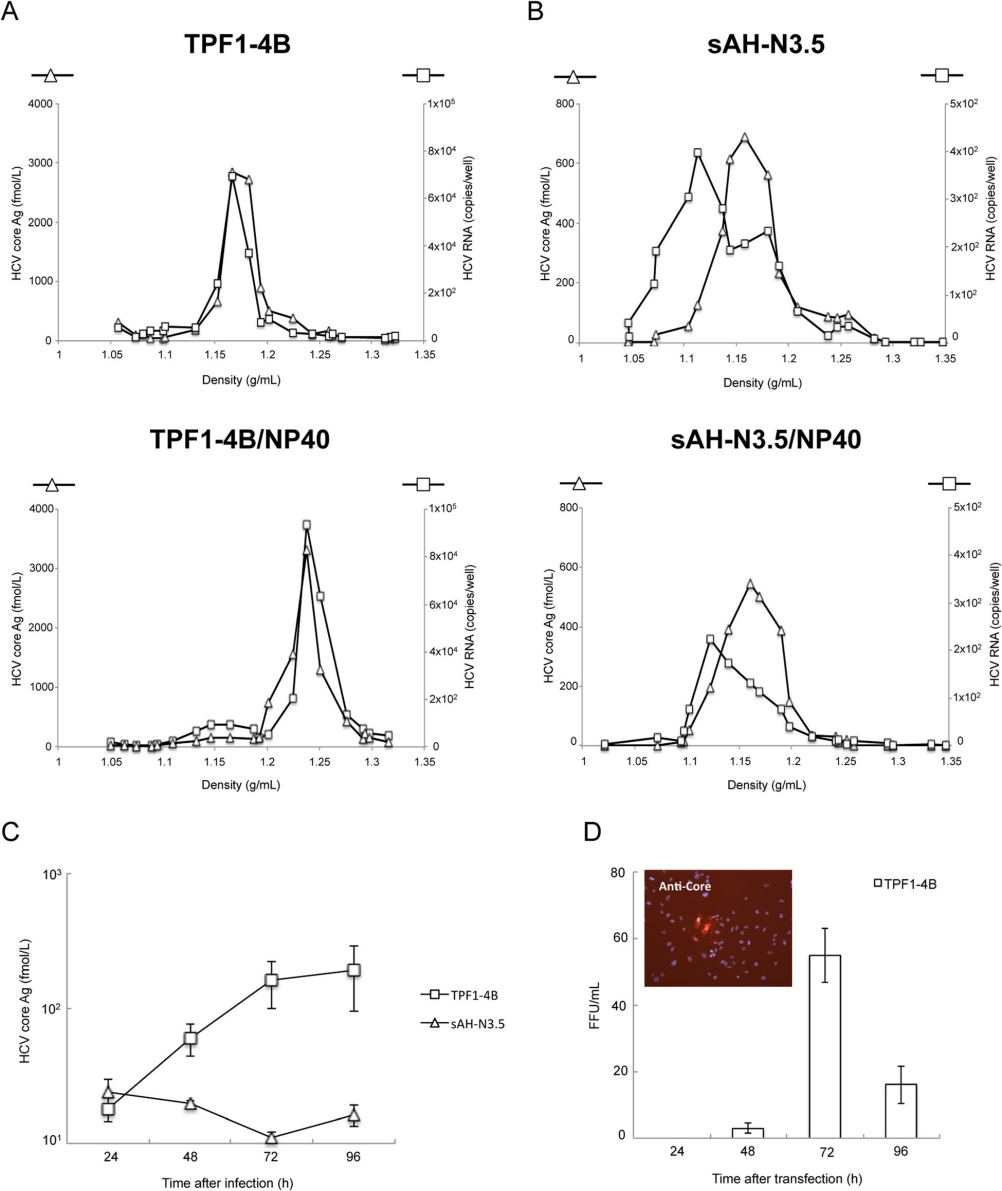
Characterization of TPF1-4B and sAH-N3.5 virus particles. **a**, **b** Concentrated culture medium collected from TPF1-4B (**a**) and sAH-N3.5 (**b**). RNA-transfected cells were fractionated using a 10–60 % stepwise sucrose density gradient. HCV RNA and core Ag levels were measured by qRT-PCR and sandwich EIA, respectively. Culture media after treatment with 0.2 % NP-40 prior to centrifugation were also analyzed (**a**, **b** lower panels). **c** Comparison of the infectivity of the culture medium from TPF1-4B RNA- and sAH-N3.5 RNA-transfected Huh7 cells. Culture media at 72 h after transfection were used to inoculate naïve Huh7 cells, and HCV core Ag levels were determined at 24, 48, 72 and 96 h after infection. **d** Time course of infectious TPF1-4B virus released into the culture medium of RNA-transfected Huh7 cells, as measured by FFA. The insert shows the HCV core Ag levels (*red*) detected by indirect immunofluorescence in cells infected with TPF1-4B viral particles at 72 h after transfection. The nuclei were counterstained with DAPI (*blue*). Assays (D and E) were conducted two independent experiments, and results represent the mean of three independent experiments ± standard deviations

The level of HCV core Ag in the culture medium obtained from cells transfected with TPF1-4B RNA was over 18-times higher than that in the medium obtained from cells transfected with sAH-N3.5 RNA (Fig. 1f) at 72 h p.t., suggesting a difference in the production of virus particles between these two HCV RNAs. We fractionated the HCV core antigens and RNA in these culture media by sucrose density gradient ultracentrifugation. The peaks of both the core Ag and HCV RNA from cells transfected with TPF1-4B RNA migrated at the same density of 1.17 g/mL (Fig. 2a, upper). Treatment of the samples with NP40 resulted in a peak shift for both the core Ag and RNA to a density of 1.25 g/mL. These observed densities of HCV RNA and core Ag were similar to those previously described for virus particles and capsids in HCVcc [5]. These data suggested that TPF1-4B could produce HCV particles in transfected cells.

In contrast, HCV RNA and the core antigen from the culture medium of sAH-N3.5-transfected cells fractionated differently (Fig. 2b, upper). The decreased amount of HCV RNA and the lack of a peak shift for the core Ag following NP-40 treatment suggested that most of the sAH-N3.5 genomic RNA was not packaged in capsids with core Ag, and thus did not form virus particles.

We examined the infectivity of the virus particles produced by monitoring the production of HCV core Ag in naïve Huh7 cells inoculated with media harvested from cells transfected with TPF1-4B or sAH-N3.5 RNA. The amount of HCV core Ag in cells inoculated with the culture medium from TPF1-4B RNA-transfected cells increased at 72 h post-infection (p.i.), whereas that in cells inoculated with the culture medium from sAH-N3.5 RNA-transfected cells did not increase (Fig. 2c). The number of HCV core Ag-positive cells (determined by a focus-forming assay [FFA]; Fig. 2d) was more than 10-times higher in the wells inoculated with the 72 h p.t. culture medium than in wells inoculated with the 48 h p.t. culture medium collected from cells transfected with TPF1-4B RNA. The lag time between the continuous secretion of core Ag into the culture medium and the abrupt increase in the number of focus-forming units (FFU; Fig. 1f) suggested that infectious virus was produced after the accumulation of core Ag. These data indicated that TPF1-4B RNA could effectively replicate and produce infectious virus in the naïve Huh7 cells.

### A sequence variant in NS2 potentiates infectious virus formation

**Fig. 3:**
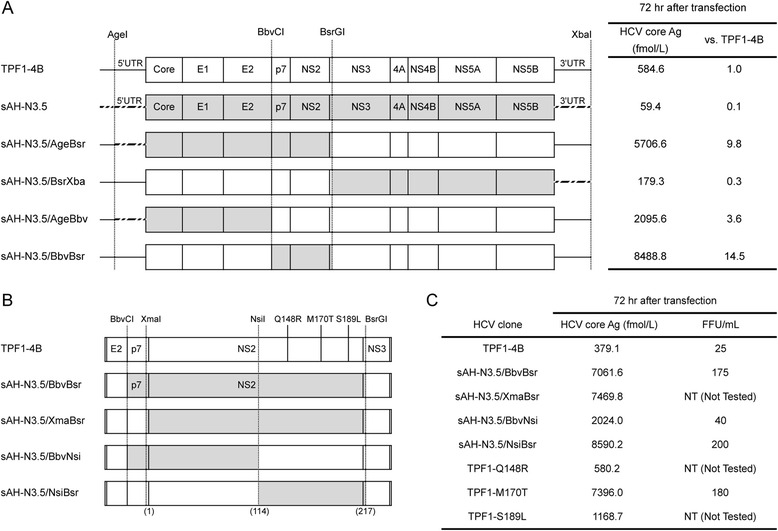
Viral particle production of chimeric sAH-N3.5/TPF1-4B virus. **a** Organization of full-length HCV constructs. Four sAH-N3.5/TPF1-4B clones were developed by swapping regions of the TPF1-4B genome (from the core to NS2, NS3 to NS5B, the core to E2, and p7 to NS2 regions) with the corresponding region of the sAH-N3.5 genome to create the sAH-N3.5/AgeBsr, /BsrXba, /AgeBbv, and /BbvBsr constructs, respectively. The sAH-N3.5 genome sequences are shown in *gray*, and the TPF1-4B sequences are shown in *white*. Huh7 cells were transfected with the full-length RNAs shown on the left, and the amounts of HCV core Ag were determined in the culture medium of cells at 72 h after transfection. **b** Organization of full-length HCV constructs. Three sAH-N3.5/TPF1-4B clones were generated by swapping the regions NS2, p7 to NS2_aa114_, and the C-terminal half of NS2 in the TPF1-4B genome with the corresponding regions of the sAH-N3.5 genome to create the sAH-N3.5/XmaBsr, /BbvNsi, and /NsiBsr constructs, respectively. The positions of the three different amino acid variants (Q148R, M170T, and S189L) were confirmed by alignment of the C-terminal half of the NS2 sequences from TPF1-4B and sAH-N3.5 (*top row*). Three different amino acids sequences were inserted into the TPF1-4B genome to create the TPF1-Q148R, TPF1-M170T, and TPF1-S189L constructs. **c** In order to compare their ability to produce viral particles, we collected the culture medium of cells at 72 h after transfection and quantified infectivity by FFA. All assays were conducted two independent experiments, and data are represented by an average

Variation in the efficiencies of subgenomic replicon replication and infectious virus production of the two different genomic RNAs suggested that some genomic segments affect the replication of genotype 1b HCV clones in the HCVcc. We investigated the segments that affect replication by exchanging segments between sAH-N3.5 and TPF1-4B (Fig. 3a). Introduction of a core-NS2 segment of TPF1-4B into the sAH-N3.5 replicon resulted in approximately 3-fold higher core Ag levels in the culture medium compared to that of the native sAH-N3.5. However, the core Ag level of this chimera was still 1/3 of that in the TPF1-4B medium, suggesting that the replication machinery of sAH-N3.5 is responsible for the poor infectious virus production.

A genomic RNA, sAH-N3.5/AgeBsr, which carried the structural regions (from the 5′UTR [*Age*I] to the NS2) of sAH-N3.5 and the nonstructural regions (from NS3 to the 3ʹUTR) of TPF1-4B, produced nearly 10-times more core Ag in the culture medium of transfected cells than the original construct (TPF1-4B) (Fig. 3a). Experiments using sAH-N3.5/AgeBbv and sAH-N3.5/BbvBsr, which carried portions of the sAH-N3.5 structural regions, showed that the region spanning from p7 to the end of NS2 strongly potentiated core Ag secretion, with levels 14.5-times greater than that of the native TPF1-4B RNA.

Among the cells transfected with the four HCV genomic RNAs carrying smaller portions of the p7-NS2 segment, sAH-N3.5/NsiBsr-transfected cells secreted the highest core Ag levels into the culture medium (Fig. 3b, c). We performed an FFA using the 72-h p.t. culture medium, and found that the culture medium of AH-N3.5/NsiBsr-transfected cells produced 8-times more foci than the medium of TPF1-4B-transfected cells (Fig. 3c). These results indicate that the C-terminal half of NS2 in sAH-N3.5 is responsible for potentiating infectious virus formation in the TPF1-4B genomic context.

There are three differences in the NS2 amino acid sequences between TPF1-4B and sAH-N3.5: Q148R, M170T, and S189L. We introduced each variant individually into TPF1-4B and assessed the infectious virus-forming activity of genomic RNAs carrying these variants. The secretion of core Ag was increased slightly in TPF1-Q148R and TPF1-S189L RNA-transfected cells compared to that of wild-type TPF1-4B. However, the secretion of the core Ag of TPF1-M170T was similar to that of sAH-N3.5/NsiBsr (Fig. 3c). In addition, the 72-h p.t. culture medium from cells transfected with TPF1-M170T produced 7-times the foci produced by TPF1-4B (Fig. 3c).

### HCV genomic RNA carrying a variant in NS2 produced infectious virus

To further analyze TPF1-M170T, we established ALS32.50-cured cells derived from Huh7 cells harboring the TPF1-4B subgenomic replicon (see Methods). ALS32.50 cells transfected with TPF1-M170T RNA secreted higher amounts of HCV RNA and core Ag into the culture medium than cells transfected with TPF1-4B RNA at 96 h p.t. (Table 2). It is noteworthy that the ratio of core Ag to HCV RNA in the culture medium of TPF1-M170T-transfected cells was lower than that of TPF1-4B-transfected cells. Inoculation of the culture medium recovered at 96 h p.t. showed that approximately 37-times more foci were produced in the TPF1-M170T culture medium than in the TPF1-4B culture medium. Using ALS32.50-cured cells, we were able to produce infectious virus particles more efficiently than using naïve-Huh7 cells.

**Table 2:** The ratio of HCV core Ag to HCV RNA and the infectivity of culture medium recovered at 96-h p.t

	Time after transfection (96 hr)					
	Core Ag		RNA	Core Ag/RNA	FFU	Specific infectiveity
	(fmol/L)	(molecules/mL)	(copies/mL)	(molecules/copy)	(/mL)	(FFU/mL)
TPF1-4B	5892.4	3.5E + 09	7.2E + 05	4926.7	35	4.9E-05
TPF1-M170T	42742.7	2.6E + 10	1.1E + 07	2427.5	1300	1.2E-04

**Fig. 4:**
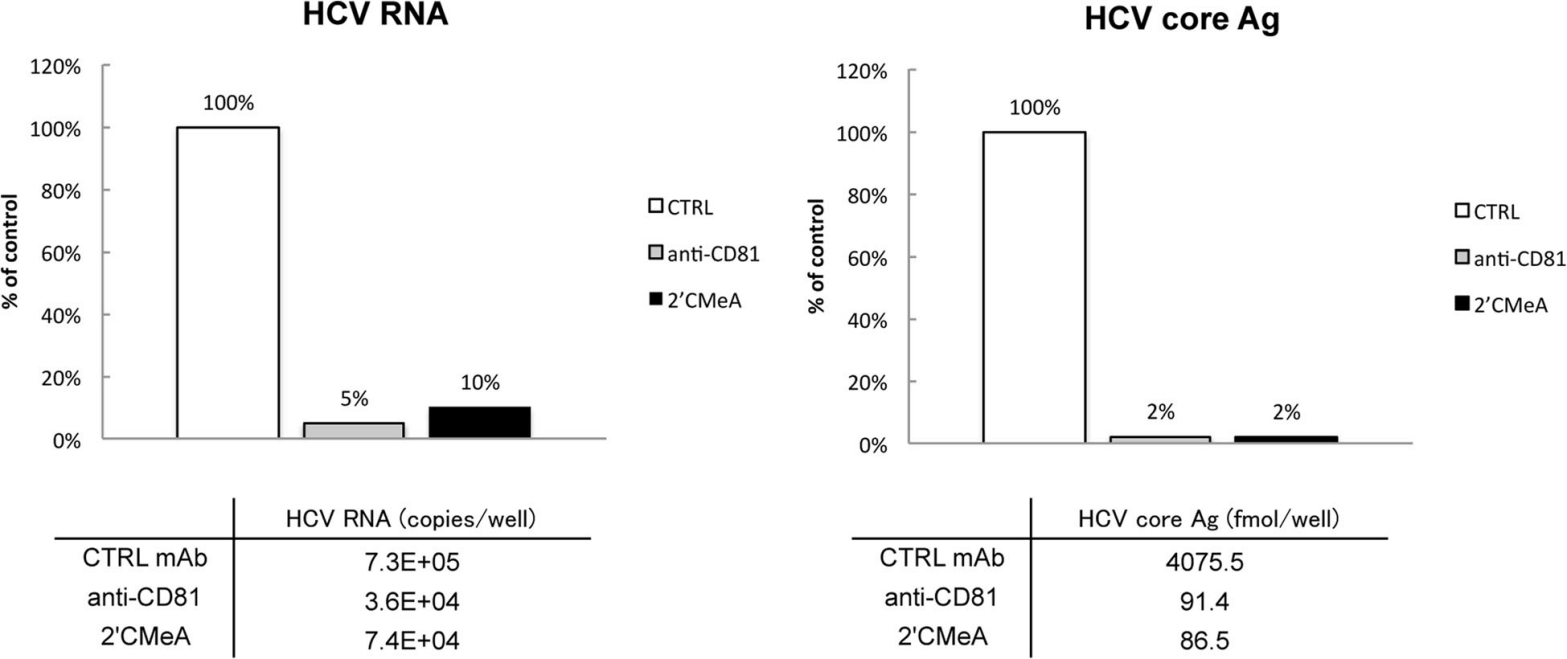
Neutralization of the cell culture by treatment with a CD81-specific antibody. ALS32.50 cells were inoculated with culture medium containing approximately 1300 FFU/mL in the presence of a CD81-specific antibody (anti-CD81, *gray bar*), polymerase inhibitor (2′CMeA, *black bar*), or a control antibody (CTRL, *white bar*), and were incubated for 96 h. Inoculated cells were analyzed by qRT-PCR and sandwich EIA. Values obtained with FFU in the presence of CTRL were set to 100 %. All assays were conducted two independent experiments, and data are represented by an average

We carried out inhibitory assays using a CD81-specific antibody and the NS5B polymerase inhibitor 2’CMeA for infection with culture medium inoculation. Using the CD81-specific antibody, we observed approximately 20-fold and 50-fold reductions of HCV RNA and HCV core Ag, respectively, in cells inoculated with the 96 h culture medium compared to cells transfected with TPF1-M170T RNA. Similar reductions in HCV RNA (10-fold) and core Ag (50-fold) were observed in cells treated with 2C’MeA (Fig. 4). These data suggested that the infectious virus produced by the cured cells transfected with TPF1-M170T RNA infected the naïve cured cells in a CD81-dependent manner, and replicated themselves using own NS5B polymerase.

## Discussion

We developed an HCVcc from genotype 1b HCV cDNA clones that were isolated from two patients with distinctive symptoms and significantly different viral RNA titers. We introduced several mutations and variants into the HCVcc to increase the efficiency of viral replication, the effects of which were sensitive to the genomic context. The established HCVcc, TPF1-M170T, could infect naïve cured Huh7 cells in a CD81-dependent manner, and replicate itself using its RdRp, NS5B.

rep-sAH-N3.5, which was derived from HCV cDNA of a patient with acute hepatitis, carried previously described adaptive mutations in NS3 [12, 15]. When these mutations were introduced into the full-length sAH-N3.5 HCV genomic RNA, the RNA efficiently replicated in Huh7-transfected cells. However, cells transfected with this viral RNA in its original full-length context barely produced any infectious particles, as the other genotype 1b clone, Con1, carrying adaptive mutations in NS3 and NS5 [31]. Though the colony forming efficiency of rep-TFP1-4B, which carries newly identified adaptive mutations in NS4B, was lower than that of rep-sAH-N3.5, TFP1-4B can produce infectious viral particles. Taking into account that Con1 genome carrying the adaptive mutations in NS4B (K1846T) can produce infectious particles with moderate interference, NS4B should have an impact on virus particle formation, in addition to the known NS4B function serving a scaffold for the viral replicase complex formation to regulate HCV genome replication [1, 40–43].

Surprisingly, the 5′-segment of rep-sAH-N3.5 could potentiate infectious virus formation in combination with 3′-half genomic segments from rep-TFP1-4B RNA carrying the adaptive mutations in NS4B not with that from rep-sAH-N3.5. In the 5′-half of the sAH-N3.5 genomic RNA, we determined that an amino acid sequence variant at the 170th position in NS2 is the variant that potentiated infectious virus formation by TPF-4B. At this position, methionine is a major variant, and M170T, which was the responsive variant in the sAH HCV genome, is rare among HCV genomes (Los Alamos HCV sequence database). The crystallographic analysis indicated that M170 is located in the crossover region of the NS2-protease dimer [44]. Alanine scanning of NS2 showed that the M170A variant exhibited increased dimer formation in vitro and that the JFH clone carrying M170A showed decreased infectivity in naïve Huh7.5 cells. However, this mutation had no significant effect on the infectivity of the J6-H77NS2-JFH chimeric HCV genome. These data indicate that the amino acid at position 170 of NS2 affects the infectivity of HCVcc in a genomic context-dependent manner.

The ratio of the core Ag to the HCV RNA levels in the culture medium of TPF1-M170T-infected cells was lower than that of TPF1-4B-infected cells, which suggested that M170T improved core protein assembly into infectious virus. Assembly deficiencies caused by mutations in core proteins have been reported to be compensated by mutations in NS3, p7, and NS2 [24]. Among these mutations, the positions of compensatory mutations in NS2 are located in the transmembrane domain. Mutations in the core, envelope proteins, p7, and NS5A, which abolish viral assembly, altered the subcellular localization of NS2 proteins [45]. The subcellular localization of NS2 is determined by its transmembrane domain. In addition, the second α-helix of the protease domain functions in the interaction between NS2 and the membrane [46]. These data suggested that M170A may affect the molecular interactions of HCV proteins, thereby changing the efficiency of capsid assembly.

Cells transfected with TPF1-4B continuously secreted core Ag into the culture medium until 96-h p.t. However, the infectivity of the medium abruptly increased at 72 h, suggesting that the accumulation of HCV proteins or induced host factors might be essential for infectious virus formation. Using cured cells, the infectivity of HCVcc was observed in the 24-h culture medium transfected with TPF1-M, and it increased through to 96 h p.t. This could be an importance of the host factor for assembly of HCVcc capsids, as previously identified in Huh7.5 cells [47, 48]. Although ALS32.50 was established by the same method as Huh7.5, another host factor might contribute the infectivity genotype 1b HCV in ASL32.50, because of the decreased infectivity of the JFH clone carrying M170A in naïve Huh7.5 cells [44]. Huh7.5 cells transfected with RNA established in this study could provide insight into a novel host factor for genotype 1b HCVcc.

Prior to the present study, no HCVcc carrying a full genotype 1b genome had been reported. Although the full-length HCV genomic RNA could replicate in cultured cells and produce infectious virus as previously reported [31, 49, 50], the infection efficiency was insufficient for autonomous virus propagation in cultured cells, even when the production efficiency increased with the inclusion of adaptive mutations. Therefore, the infectivity of the virus particles must be further improved to develop a robust HCVcc of genotype 1b. We acknowledge that the efficiency of virus replication of the present HCVcc is quite low compared to the HCVcc containing JFH-1 genomic segments. However, our system will nevertheless provide insight into genotype 1b HCV, which is the second major genotype of HCV.

## Conclusions

In conclusions, we established a novel HCVcc genotype 1b in Huh7 cells by introducing an amino acids variant in NS2 and the mutations in NS4 in HCV genomic RNA isolated from a patient with fuluminant HCV after liver transplantation.

## Additional file

Additional file 1: Table S1. List of Primers. (PDF 34 kb)

## Abbreviations

2′CMeA: 2′C-methyladenosine
AH: Acute hepatitis
EMCV: Encephalomyocarditis virus
FCH: Fibrosisng choleostatic hepatitis
FFA: Focus-forming assay
HCV: Hepatitis C virus
HCVcc: Cell-cultured hepatitis C virus
IFN: Interferon-alpha
IRES: Internal ribosome entry site
IRRDR: IFN/RBV Resistance-determining region.
ISDR: IFN Sensitivity-determining region
Neo: Neomycin phosphotransferase
NR: Non responder
RBV: Ribavirin
RdRp: RNA-dependent RNA polymerase
SVR: Sustained virologic response
UTR: Untranslated region
