# Supplementary material for: Production of infectious HCV genotype 1b virus in cell culture using a novel Set of adaptive mutations
Source: BMC Microbiol. 2016 Sep 27;16:224. doi: 10.1186/s12866-016-0846-9 (PMC5039931; doi:10.1186/s12866-016-0846-9)
Supplement: Additional file 1: Table S1. — List of Primers. (PDF 34 kb) [file 12866_2016_846_MOESM1_ESM.pdf]

Table S1. List of Primers.

Primer	Sequence (5'-3')
1b5290AS	GACATGCATGTCATGATGTATTTG
1b9405R[3)]	GCCTATTGGCCTGGAGTGTTTAGCTC
3UTR-1F	ATCTTAGCCCTAGTCACGGC
5'RACE Abridged Anchor primer	GGCCACGCGTCGACTAGTACGGGIIGGGIIGGGIIG
Adp	CTAGACTCGAGTCGACATCG
chiba-S[1)]	TAGTGGTCTGCGGAACCGGT
chiba-AS[1)]	TGCACGGTCTACGAGACCT
dT-Adp	CTAGACTCGAGTCGACATCGTTTTTTTTTTTTTTTTT
EMCV-S1	TGCACATGCTCTACATGTGTTTAGTCGAGG
HC85F[1)]	ATGGCGTTAGTATGAGTGTCGTGCAGCCT
HC4498S	AGGGGGGGAGGCATCTCATTTTCTG
HC4888F	TGCTATGACGCGGGCTGTGCTTGGTA
HC8939F[1)]	CTACGGGGCCTGTTACTCCATTGAAC
HC9302R[1)]	TCGGGCACGAGACAGGCTGTGATATATGTCT
HC-Long A1[1)]	ATCGTCTTCACGCAGAAAGCGTCTAGCCAT
KM2	AGGCATTGAGCGGGTTTAT
KY78	CTCGCAAGCACCCTATCAGCCAGT
Universal Amplification primer	CUACUACUACUAGGCCACGCGTCGACTAGTAC
XR58F	CTAGCTGTAAAGGTCCGTGAGCCGCATGA
XR58R[2)]	TCATGCGGCTCACGGACTTTCACAGCTAG

[1)] Yagi et al. [32]

[2)] Yanagi et al. [33]

[3)] Yanagi et al. [34]
